# Overview of Sodium-Glucose Co-transporter 2 (SGLT2) Inhibitors for the Treatment of Non-diabetic Heart Failure Patients

**DOI:** 10.7759/cureus.17118

**Published:** 2021-08-12

**Authors:** Irina Balan, Tetyana Khayo, Sevara Sultanova, Yuliia Lomakina

**Affiliations:** 1 Internal Medicine, Nicolae Testemițanu State University of Medicine and Pharmacy, Chisinau, MDA; 2 Obstetrics and Gynecology, Bukovinian State Medical University, Chernivtsi, UKR; 3 Cardiology, Tashkent Pediatric Medical Institute, Tashkent, UZB; 4 Medical Biology and Genetics, Bukovinian State Medical University, Chernivtsi, UKR

**Keywords:** chronic heart failure, cardiovascular outcomes trials, a sodium-glucose cotransporter 2 inhibitor, empagliflozin, canagliflozin, dapagliflozin

## Abstract

Heart failure (HF) is a clinical syndrome that results from any structural or functional impairment of ventricular filling or ejection of blood, and results in low life quality and expectancy, creating a significant burden on the healthcare system. The pharmacological HF management has remained unchanged for a decade, however, several randomized clinical trials have demonstrated the potential clinical benefits of sodium-glucose cotransporter-2 inhibitors, an antidiabetic agent, by reducing the rate of hospitalizations for HF, cardiovascular death, and all-cause death. The cardioprotective effects are characterized by reduction of inflammatory, metabolic and ionic dyshomeostasis despite the diabetic status. Since the United States Food and Drug Administration (FDA) approval in May 2020, SGLT2 inhibitors have been used mostly in heart failure with reduced ejection fraction (HFrEF). In this review article, we provide a comprehensive overview of the potential benefits, effectiveness, and safety profile of SGLT2 inhibitors used in HF patients with no history of diabetes mellitus.

## Introduction and background

Heart failure (HF) is a clinical syndrome that comprises fatigue, dyspnea, reduced exercise tolerance, and fluid retention as a result of a deterioration of ventricular filling and/or ejection fraction [1]. According to the United States Centers for Disease Control and Prevention (CDC), approximately 6.5 million adult Americans were affected by heart failure causing one in eight deaths in 2017 [2]. HF is one of the leading causes of hospital readmissions and creates huge stress on the healthcare system [3]. Worldwide, 1-2% of the adult population lives with heart failure, which is accompanied by reduced quality of life, high morbidity, mortality, and significant financial burden [4]. HF causes a high prevalence of early post-discharge re-hospitalization and mortality, which causes a major ordeal to public health. The need for new pharmacological interventions has been reinforced by the constant threat to the morbidity and mortality caused by heart failure [2, 3].

The pharmacological HF management has remained relatively unchanged for a decade and included beta-blockers, diuretics, and renin-angiotensin system (RAS) inhibitors [5]. Regardless of the major cardiovascular benefits of these agents, patients still have an increased risk for morbidity and mortality [6]. In 2017, the United States Heart Failure with reduced Ejection Fraction (HFrEF) guideline update was implemented by the addition of combined angiotensin receptor-neprilysin inhibitor sacubitril-valsartan to a list of pharmacologic agents recommended for use in the treatment of heart failure [7]. A new approach to pharmacological treatment of HF was implemented in May 2020 with the approval of sodium-glucose co-transporter 2 (SGLT2) inhibitors (dapagliflozin) by the FDA for the treatment of adults with heart failure with reduced ejection fraction with and without type 2 diabetes mellitus (T2DM) to mitigate the risk of cardiac mortality and admissions due to HF and associated disorders [8].

As a group of active glucose transporter proteins, SGLT2 is mainly expressed in the kidneys [9]. SGLT2 inhibitors promote the osmotic diuresis and natriuresis from proximal convoluted tubules by inhibiting the absorption of glucose and sodium, leading to a reduction of the cardiac preload, which thus causes a decrease of the arterial blood pressure and afterload, thereby improving the subendocardial circulation [4]. There is a multitude of clinical trials investigating the potential use of SGLT2 inhibition in HF patients with and without T2DM [4]. At this time the effectiveness and safety profile of SGLT2 inhibitors in patients with chronic heart failure without diabetes mellitus remains incompletely understood [10].

The objective of our study is to provide a comprehensive review of the potential benefits, effectiveness, and safety profile of SGLT-2 inhibitors in HF patients with no history of diabetes mellitus and to summarize the most relevant clinical evidence which supports the rationale for their use in HF patients without T2DM.

Methods

A comprehensive literature review was performed collecting the articles from PubMed, Google Scholar, and ScienceDirect databases using medical subject headings (MeSH) keywords: heart failure, sodium-glucose cotransporters-2 inhibitors, and nondiabetic patients. The following inclusion criteria were applied: published in English only, clinical trials and review articles involving human subjects, and articles involving animal subjects for preclinical research. The collection of the articles for the study was done ethically.

## Review

The novel antidiabetic agents, SGLT2 inhibitors, have been gradually reconsidered as an important class of medications for heart failure, being recommended for their beneficial impact in patients with type 2 diabetes patients and multiple cardiovascular risk factors or heart failure with reduced ejection fraction [11]. The current clinical guidelines for the management of heart failure are concentrated on a triple neurohormonal blockade, including beta-blockers, angiotensin-converting enzyme inhibitors (ACEIs), or angiotensin receptor blockers (ARBs), and mineralocorticoid receptor antagonists with specific recommendations based on the patient’s comorbidities [12]. 

SGLT2 inhibitors shown to improve the clinical outcomes in the prevention of heart failure in patients with T2DM [13]. The underlying protective cardiovascular and renal mechanisms of this drug class in patients with or without T2DM have promising benefits for the treatment of both heart failure with reduced and preserved ejection fraction [13]. In February 2021, Chambergo-Michilot et al. published a comprehensive systematic review and meta-analysis that showed the multiple benefits of SGLT2 inhibitors in HFrEF patients compared to the placebo, yet their mechanism has not been conclusively determined [12]. 

Direct cardiac effects of SGLT2 inhibitors

SGLT2 Inhibitors' Effects on Cardiac Inflammation

The research performed by M. Rauchlaus and his colleagues has revealed the correlation between systemic inflammation and the progression and severity of chronic heart failure [14]. Multiple inflammatory markers, such as soluble CD14 receptor (sCD14), interleukin 6 (IL-6), tumor necrosis factor ɑ (TNF-ɑ), soluble TNF receptor 1 and 2 (sTNF-R1/sTNF-R2), could have an impact on the prognosis of HF [14]. In 1998, Oyama et al. concluded that the impaired nitric oxide and oxidative stress pathways are responsible for cytokine-induced left ventricle (LV) dysfunction, and impact various cells, including endotheliocytes, vascular smooth muscle cells, infiltrating inflammatory cells, and cardiomyocytes [15]. Based on these pathophysiological mechanisms, anti-inflammatory and anti-cytokine therapies could be considered beneficial, although the clinical trials have not significantly improved the survival of patients with HF [11].

Several experimental studies have been focused on the use of SGLT2 inhibitors in HF with T2DM [9]. For example, empagliflozin can reduce the cardiac macrophage infiltration in genetic/diabetes mouse model; or dapagliflozin improves the cardiac inflammation in Wistar rats by elevating the anti-inflammatory messenger RNA (mRNA) levels (IL10), attenuating the inflammatory cytokines mRNA levels (IL-1𝛃 and IL-6), and inducing the macrophage polarization to an anti-inflammatory phenotype [9]. Further to these preclinical researches, a recent meta-analysis published in 2020 has revealed that SGLT2 inhibitors decrease the serum level of C-reactive protein (CRP), IL-6, and TNF-ɑ, and, subsequently, inhibit the pro-inflammatory signaling pathways [11]. 

SGLT2 Inhibitors' Effects on Cardiac Oxidative Stress

Oxidative stress is defined by the overproduction of reactive oxygen species (ROS) caused by mitochondrial dysfunction, increased activity of nicotinamide adenine dinucleotide phosphate (NADPH) oxidase, and nitric oxide synthase combined with insufficient endogenous antioxidants [11]. Considerable experimental and clinical studies have demonstrated that ROS enhances the injury by non-specific interaction with proteins, lipid membranes, mitochondria, and DNA resulting in severe contractile dysfunction and heart failure [16].

Animal models have demonstrated that SGLT2 inhibitors exhibit antioxidant properties by reducing cardiac oxidative stress, and antihyperglycemic effects [9]. The research of Kusaka H. and his colleagues showed that a 10-week empagliflozin regimen in the prediabetes/metabolic syndrome rat model considerably decreases superoxide levels in the myocardium with no autonomic dysfunction improvement or blood pressure reduction [17]. Because oxidative stress and inflammation have a crucial impact on the pathogenesis of cardiac hypertrophy and remodeling, this report also validates that empagliflozin attenuates these effects and ameliorates the hypertrophic and fibrotic processes [17]. A recent systematic review has concluded that SGT2 inhibitors are involved in reducing the levels of some essential biomarkers of oxidative stress, prostaglandin-F2ɑ and 8-hydroxy-2′-deoxyguanosine [11]. Despite the mentioned benefits of SGLT2 inhibitors, further studies are needed to understand the precise mechanism of their cardioprotective effects.

SGLT2 Inhibitors' Effects on Cardiac Energy Metabolism

Hypoxia could induce the shift of the glucose metabolism to anaerobic glycolysis, resulting in the reduction of adenosine triphosphate (ATP) production [11]. Initially, cardiac disease is associated with adaptation to compensate and decrease the injuring processes, however, chronic activation will lead to decompensation and the progression of heart failure [18]. 

SGLT2 inhibitors induced glycosuria, and hypoglycemic effects, stimulates endogenous (hepatic) glucose production (EGP), fat oxidation, lipolysis, ketogenesis, and reduces total glucose dissociation (TGD) and glucose oxidase activity (GOx) [19]. Hence, glycosuria shifts the whole-body metabolism to lipid mobilization and utilization, and this pattern is not specific only to diabetic patients [19].

SGLT2 Inhibitors' Effects on Cardiac Ionic Homeostasis

The cardiomyocytes' homeostasis of calcium ion (Ca^2+^) involves the influx through mitochondrial Ca^2+^ uniporter (MCU) and the efflux through mitochondrial Na^+^/Ca^2+^ exchanger (mNCE) [18]. The disruption of this pathway is determined by cytoplasmic sodium ion (Na^+^)^ ^overload and induces the reduction of nicotinamide adenine dinucleotide phosphate [NAD(P)/NAD(P)^+^] redox potential and the enhancement of oxidative stress [20]. Ting Liu et al. showed on the guinea pig model, the impact of the insufficient mitochondrial Ca^2+ ^metabolism in the heart failure progress and the associated complication of sudden cardiac death [20].

Regardless of the lack of SGLT2 expression on cardiomyocytes, empagliflozin has been involved in reducing the cytoplasmic Na^+^ and Ca^2+^ loads and increasing the mitochondrial Ca^2+^ by the direct inhibition of Na^+^/hydrogen ion (H^+^) exchange (NHE) [9]. It has been proven that SGLT2 inhibitors, such as dapagliflozin and empagliflozin, increase the activity of sarcoplasmic endoplasmic reticulum Ca^2+^-ATPase (SERCA2a) responsible for the Ca^2+^ reuptake and, therefore, leading to the improvement of the left ventricular diastolic function [9]. The effects of SGLT2 inhibitors on cardiac ionic dyshomeostasis have been mentioned in the findings of the EMPA-REG OUTCOME trial [9].

Renoprotective Effects of SGLT2 Inhibitors in Heart Failure (HF)

Recent clinical trials indicated that SGLT2 inhibitors are efficient in preventing heart failure in patients with or without diabetes due to the combined cardiorenal protective mechanisms [14]. SGLT2 inhibitors can interfere with the glucose and Na^+ ^reabsorption in the proximal convoluted tubule, and increase the natriuresis in the late straight segment of the proximal tubule [11]. Consequently, these processes enhance the concentration of sodium in the distal macula, and diminish intraglomerular hypertension and hyperfiltration, resulting in ameliorated glomerular afferent arteriolar regulation and cardiorenal protective effects [11].

The systemic effects caused by SGLT2 inhibition on the kidney have been studied mostly in diabetic patients enrolled in EMPA-REG OUTCOME and CANVAS trials, which revealed an approximately 40-50% reduction of the relative risk and hazard ratio for estimated glomerular filtration rate (eGFR) declining [12]. Recent preclinical research performed in the Netherlands aimed to determine the effects of SGLT2 inhibition in nondiabetic rats with LV dysfunction after myocardial infarction [21]. It was concluded that empagliflozin acts as a profound diuretic without any effects on renal structure or function or ionic dyshomeostasis with no evidence of increased bone mineral resorption [21].

Clinical Benefits of SGLT2 Inhibitors in HF

A recent meta-analysis of several clinical trials, which involved approximately 17,000 individuals, has concluded that SGLT2 inhibitors markedly reduce heart failure hospitalizations (HFH), adverse renal effects, and HF mortality consistently in diabetic and nondiabetic patients [22]. Globally, despite the progress of the HF treatment, this condition remains one of the main causes of cardiovascular morbidity and mortality and is the leading cause of hospitalizations in seniors in the United States with 9.3% of cardiovascular-related deaths being attributed to HF [12]. The recent clinical trials indicated that the hypoglycemic agents have the potential to prevent HF and adverse events associated with the disease [12, 23], specifically the SGLT2 inhibitors’ use in preventing HF-related events [23].

The potential benefits of SGLT2 inhibitors for patients with HF have been identified by several randomized clinical trials, such as the DECLARETIMI 588 trial, the CANVAS program, and the EMPA-REG OUTCOME [12, 23]. The hospitalizations for HF declined significantly following treatment with SGLT2 inhibitors [23]. Moreover, emergency room visits, all-cause mortality, and cardiovascular mortality due to HF declined with SGLT2 inhibitors use [12]. The DAPA-HF trial showed that dapagliflozin has beneficial effects on reducing the prevalence of HF hospitalizations, subsequently of the rate of cardiovascular mortality, despite the diabetes status [23]. The findings of the trial suggested that glycemic control does not interfere with the intervention’s efficacy [12, 23].

While the mechanism of action of the SGLT2 inhibitors remains a matter of active research, extant research provides useful insight on the topic [12, 24]. The cardioprotective effects of the SGLT2 inhibitors extend beyond glycemic control in patients with HF [12, 24]. The metabolic impact of these agents are associated with the elevation of ketone body production, activation of anti-inflammatory and antioxidative pathways, and reduction of advanced glycation end-products mediated effects [24, 25] Therefore, the mechanism of action of the SGLT2 inhibitors is complex and multifactorial [12, 25]. While these findings justify the administration of SGLT2 inhibitors for patients with HFrEF and diabetes, recent trials reported comparable benefits in nondiabetic HF patients [10]. The FDA approved dapagliflozin use in both diabetic and non-diabetic adult HF patients [10]. The pharmacologic intervention decreased the preload due to natriuresis and diuresis, and the afterload due to reduction of arterial blood pressure and changing vascular function [10].

The aforementioned trials had shown that the reduced hospitalizations and cardiovascular deaths in non-diabetic HF patients are not limited to the initial recommendations [10] and the improved survival rate without increasing the risk of adverse reactions [22, 24]. Chambergo-Michilot et al. indicated that the SGLT2 inhibitors cause a significant reduction in serious adverse events [12]. However, the need for monitoring renal function in patients with chronic kidney disease should be considered as the SGLT2 inhibitors are associated with increased hematocrit and urinary tract infections [12]. In this case, safety remains an area of concern and further investigations are needed [12].

DAPA‐HF is the primary trial proving a critical advantage of dapagliflozin in HFrEF with a significant drop in hospitalizations due to the cardiovascular complications and exacerbation of heart failure amongst patients with HFrEF (HR 0.74; 95% CI 0.65-0.85) [25]. The analysis of the DAPA-HF trial has shown the benefits of dapagliflozin in mitigating the risk of heart failure progression and cardiac mortality, emphasizing the importance of early therapy initiation in all hospitalized patients with HFrEF [26]. The DAPA-HF trial indicated a decline in the risk of heart failure exacerbation or cardiovascular mortality in the dapagliflozin group compared to the placebo group with a 55% efficiency in nondiabetic patients [27]. The safety of the intervention was characterized by a low number of patients discontinuing dapagliflozin due to adverse events [27]. The findings of the trial had proved that dapagliflozin could be considered an important additional treatment for HF patients regardless of their glycemia [27].

The EMPEROR-Reduced trial revealed a reduction in cardiovascular deaths and exacerbation of HF with HFrEF (HR 0.75; 95% CI 0.65-0.86) [25] and had demonstrated the impact of empagliflozin on lowering the HFH and the risk of cardiovascular mortality compared to the placebo group regardless of diabetes status [28]. However, the EMPEROR-Reduced trial involved patients with more serious GFR and LV systolic dysfunction [25]. Correspondingly, the data from this trial indicated that empagliflozin induced a decline in the risk of HF admissions and cardiovascular death despite the diabetes status [28], and its efficacy is demonstrated by the slowed decline in renal function [29]. 

## Conclusions

SGLT2 inhibitors have become an important novel class of drugs for heart failure, due to several favorable hemodynamic and metabolic effects, and are used in patients with HFrEF despite the diabetic status. The ongoing or upcoming clinical studies should further evaluate the promising benefits of SGLT2 inhibitors in heart failure with preserved ejection fraction and acute heart failure. 
